# Acute kidney injury following on-pump or off-pump coronary artery bypass grafting in elderly patients: a retrospective propensity score matching analysis

**DOI:** 10.1186/s13019-020-01226-8

**Published:** 2020-07-24

**Authors:** Rui Wang, Xian Wang, Yifan Zhu, Wen Chen, Liangpeng Li, Xin Chen

**Affiliations:** 1 Department of Cardiovascular Surgery, Nanjing First Hospital, Nanjing Medical University, Nanjing, 68 Changle Rd, Nanjing, 210006 People’s Republic of China; 2 Department of Laboratory Medicine, Nanjing Drum Tower Hospital, Nanjing University Medical School, Nanjing, 321 Zhongshan Rd, Nanjing, 210008 People’s Republic of China

**Keywords:** Acute kidney injury, Coronary artery bypass grafting, Elderly, Cardiopulmonary, Glomerular filtration rate, Long-term survival

## Abstract

**Objectives:**

This single-centre, retrospective propensity score matching (PSM) study designed to study the impact of cardiopulmonary bypass (CPB) on postoperative acute kidney injury (AKI) and the relationship between AKI and long-term outcomes in elderly patients undergoing coronary artery bypass grafting (CABG).

**Methods:**

After PSM, 466 pairs of patients (A group, on-pump; B group, off-pump) who were aged≥70 years undergoing first isolated CABG surgery from January 2012 to December 2016 entered the study. AKI was defined and classified according to the Acute Kidney Injury Network (AKIN) criteria. The incidence and severity of in-hospital AKI were compared. The impacts of AKI on the long-term outcomes including new onset of dialysis and mortality were analyzed.

**Results:**

The two PSM groups had similar baseline and procedure except whether the CPB was used or not. In hospital and 30-day mortality was of no difference(χ2 = 0.051, *p* = 0.821). AKI of any severity occurred in 40.3% of all patients, with stage 1 accounting for most cases. No difference regarding the incidence and severity of AKI could be found: AKIN stage 1: 139 (29.8%) vs 131 (28.1%); AKIN stage 2: 40 (8.6%) vs 35 (7.5%); AKIN stage 3: 18 (3.9%) vs 13 (2.8%), (u = 0.543, *p* = 0.532). No difference was observed in the in-hospital new onset of dialysis (χ2 = 0.312, *P* = 0.576). The use of CPB was not found to influence long-term new onset of dialysis (χ2 = 0.14, *p* = 0.708) and mortality (χ2 = 0.099, *p* = 0.753). Comparing with non-AKI patients, AKI patients were associated with an increased rates of new onset of dialysis (χ2 = 8.153, *p* = 0.004) and mortality (χ2 = 6.277, *p* = 0.012) during the follow-up. Multivariable logistic regression manifested that the HR for long-term new onset of dialysis and mortality in AKI patients vs. non-AKI patients was 1.83 and 1.31 respectively (95%CI 1.12–2.86, *p* = 0.007; 95%CI 1.17–2.58, *p* = 0.015). The HR for long-term new onset of dialysis and mortality in on-pump group vs. off-pump group was 1.07 and 1.11 respectively (95%CI 1.03–1.23, *p* = 0.661; 95%CI 1.09–1.64, *p* = 0.702).

**Conclusions:**

For elderly CABG patients, AKI was common, but deterioration of dialysis was a seldom incidence. Comparing with on-pump, off-pump did not decrease the rates or severity of AKI, long-term new onset of dialysis or mortality. AKI was associated with an increased long-term new onset of dialysis and decreased long-term survival.

## Background

Acute kidney injury (AKI) is a sudden loss of kidney function defined by an acute increase in serum creatinine concentration and decrease in urinary output [1]. Up to 30% of patients with varying severity develop AKI after coronary artery bypass grafting (CABG) surgery, and approximately 2% require temporary dialysis [2]. Postoperative AKI is associated with increased short and long term morbidity and mortality [3, 4].

Based on the presence of cardiopulmonary bypass (CPB) or not, CABG could be divided into on-pump CABG and off-pump CABG. It is generally accepted that CPB is a risk factor for AKI after CABG because that CPB is associated with inflammatory response, nonpulsatile flow, hemodilution, renal hypoperfusion, atheroembolism, and free hemoglobin [5, 6]. However, whether off-pump technique could improve the outcome of CABG is still a controversial issue, and many studies have compared outcomes between on-pump and off-pump technique. In general, there is a higher proportion of AKI in the on-pump group than in the off-pump group, [7, 8] but no significant differences with regard to the outcomes of mortality and renal failure [7, 9, 10]. These results manifested that postoperative AKI in the on-pump CABG did not translate to adverse outcomes, which conflicted with results from the literature conducted previously. Furthermore, age is another risk factor for AKI, but studies focused on the elderly(aged≥70 years) patients who are especially vulnerable to AKI undergoing CABG are relatively rare.

The purpose of this study was to study AKI in elderly patients undergoing first isolated CABG performed with either on-pump or off-pump technique through a single centre, retrospective propensity score matching(PSM) study. Compared with on-pump, we speculated off-pump CABG might reduce the incidence and magnitude of AKI in vulnerable elderly patients, and if correct, aimed to obverse if there was a difference in long-term kidney function and survival.

## Methods

### Definition of renal function

The glomerular filtration rate (GFR) was calculated by the abbreviated Modification of Diet in Renal Disease equation:186 × (serum creatinine/88.4)^-1.154^ × (age)^-0.203^ × (0.742 if female). Kidney function before CABG was graded from I to V according to the GFR as proposed by the Kidney Disease Outcome Quality Initiative [11]. Patients with preexisting stage V(end-stage kidney failure with a GFR below 15 or renal replacement) were excluded, and patients suffered from any nephrotoxicity were also excluded from further analysis postoperatively.

AKI was defined and classified according to the criteria proposed by the Acute Kidney Injury Network (AKIN) as AKIN stage 1: increase creatinine × 1.5 from baseline or increase of > 0.3 mg/dL within 48 h; AKIN stage 2: increase creatinine × 2 from baseline; and AKIN stage 3: increase in creatinine × 3 from baseline or creatinine > 4 mg/dL with an acute increase > 0.5 mg/dL within 48 h or new-onset of dialysis therapy [12].

### Study population

A standard set of perioperative data was collected prospectively for all patients undergoing first isolated CABG at Nanjing First Hospital between January 2012 to December 2016. Clinical data were retrospectively collected from Jiangsu Province CABG registry study database. The register website is: http://221.226.218.114:10004/Multicenter. Patients undergoing a concomitant cardiac surgical procedure, reoperation, urgent or emergent operation, minimal invasive operation, or with incomplete information were excluded. This study was approved by The Ethics Committee of Nanjing First Hospital. All patients agreed that their stored material was enclosed and that their clinical data were anonymously used for statistical analysis.

All CABG surgeries were performed by the experienced surgeons. On-pump CABG was performed via median sternotomy using a membrane oxygenator equipped with an arterial filter, cold blood antegrade cardioplegia under moderate systemic hypothermia (30–34 °C); hematocrit was maintained about 20–22% during CPB. Off-pump CABG was performed with a stabilizator and an intra-coronary shunt.

Totally there were 1358 cases up to the standard for analysis. To control the selection bias in the comparison among on-pump group and off-pump group, a propensity score (PS) analysis was performed. One PS was calculated for each patient by means of logistic-regression analysis using 16 preoperative and surgical variables including: age, gender, body mass index(BMI), diabetes mellitus(DM), hypertension, hyperlipemia, GFR, chronic obstructive pulmonary disease(COPD), history of cerebral and myocardial infarction(MI), history of percutaneous coronary intervention(PCI), left ventricular ejection fraction(LVEF), number of vessel disease, Euro-SCOREII, number of distal anastomosis, the application of left internal mammary artery(LIMA) and radial artery. Every on-pump CABG patient was matched with an off-pump CABG patient with the closest PS (within0.030). Finally, 466 pairs were successfully built in a 1:1 manner through matching PS [A group (on-pump, *n* = 466) and B group (off-pump, *n* = 466)].

### Statistical analysis

Data are represented as the mean ± standard deviation unless otherwise indicated. Categorical variables are represented as frequency distributions and single percentages. Normally distributed continuous variables were compared using a Student t-test, non-normally distributed continuous variables using the Mann-Whitney U test, and categorical variables were compared by χ2 test. Potential risk factors were calculated by Cox regression analysis. Potential independent predictors of outcome were identified by univariate Cox regression analysis. All statistical tests were two-sided. A *p*-value of less than 0.05 was considered significant. All statistical analyses were done with IBM SPSS Statistics 20.0 or STATA Data analysis and statistical software.

## Results

### Matching of patients

Matching data from A group(*n* = 466) and B group (*n* = 466) were analyzed. There were no significant differences with regard to age, gender, BMI, DM, hypertension, hyperlipemia, GFR, COPD, history of cerebral and MI, history of PCI, LVEF, number of vessel disease, Euro-SCOREII, number of distal anastomosis including LIMA and radial artery. (Table 1).

**Table 1:** Baseline and procedural characteristics after matching

variable	A group	B group	*p* value
	(*n* = 466) No. (%)	(*n* = 466) No. (%)	
Female gender	84(18.0)	89(19.1)	0.736
Age, y	73.2 ± 7.2	74.0 ± 8.4	0.119
Body mass index, kg/m^2^	25.6 ± 4.3	25.1 ± 4.7	0.091
DM			
Insulin-dependent	74(15.9)	67(14.4)	0.583
Non-insulin-dependent	68(14.6)	61(13.1)	0.569
Hypertension	246(52.8)	237(50.9)	0.6
Hyperlipemia	137(29.4)	123(26.4)	0.342
GFR(ml/min/1.73m^2^)	81.3 ± 16.4	80.4 ± 15.7	0.392
COPD	38(8.2)	44(9.4)	0.563
Previous			
Cerebal infarction	24(5.2)	29(6.2)	0.572
Myocardial infarction	75(16.1)	66(14.2)	0.465
PCI	81(17.4)	73(15.7)	0.537
LVEF			0.33
> 0.50	363(77.9)	375(80.5)	
0.30–0.50	98(21.0)	87(18.7)	
< 0.30	5(1.1)	4(0.9)	
Extent of CAD			0.187
1 vessel	17(3.6)	25(5.4)	
2 vessel	48(10.3)	54(11.6)	
3 vessel	401(86.1)	387(83.0)	
LM	142(30.1)	122(26.2)	0.167
EuroScoreII	2.6 ± 1.2	2.5 ± 1.3	0.223
Distal anastomosis	3.4 ± 0.9	3.3 ± 1.1	0.129
LIMA	438(94.0)	444(95.3)	0.467
Radial Artery	72(15.5)	63(13.5)	0.457
CPB time(min)	78.4 ± 21.7	–	–

*DM* diabetes mellitus, *PCI* percutaneous coronary intervention, *COPD* chronic obstructive pulmonary disease, *GFR* glomerular filtration rate, *LVEF* left ventricular ejection fraction, *CAD* coronary artery disease, *LIMA* left internal mammary artery, *CPB* cardiopulmonary bypass

### In-hospital outcomes

**Fig. 1:**
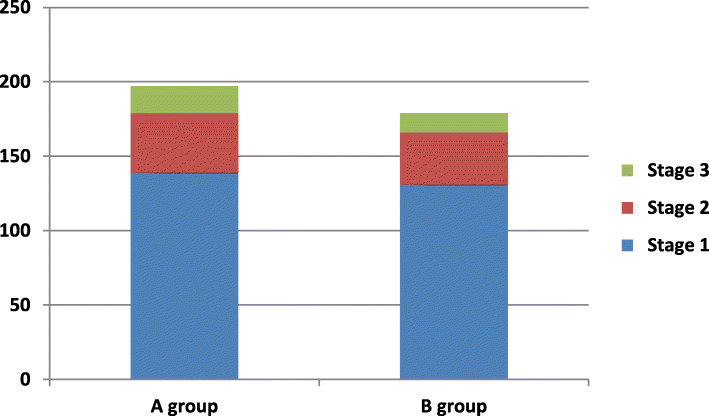
Acute kidney injury (AKI), as defined and classified according to the criteria proposed by the Acute Kidney Injury Network (AKIN), is shown stratified according to kidney function at baseline (blue = AKI 1, red = AKI 2, green = AKI 3; y-axis = number of patients)

A total of 20 patients died after the operation (11 in A group and 9 in B group, *p* = 0.821). There were no significant differences with regard to in-hospital mortality, MI, stroke, respiratory failure, pneumonia, AKI, in-hospital dialysis, RBC transfusion, deep sternal wound infection (DSWI), low cardiac output syndrome(LCOS), intra-aortic balloon pump(IABP) support. AKI of any severity occurred in 40.3% patients totally (197 in A group and 179 in B group), with AKIN stage 1 accounting for most of the AKI cases(71.8%).No significant difference in the rate and severity of AKI was found between the two groups.(u = 0.543, *p* = 0.532). (Table 2), (Fig. 1).

**Table 2:** Postoperative outcomes in the matched cohort

variable	A group	B group	*p* value
	(*n* = 466) No. (%)	(*n* = 466) No. (%)	
In-hospital			
Mortality	11(2.4)	9(1.9)	0.821
MI	19(4.1)	15(3.2)	0.6
Stroke	11(2.4)	7(1.5)	0.475
Respiratory failure	20(4.3)	15(3.2)	0.491
Pneumonia	26(5.6)	21(4.5)	0.549
AKI	197	179	0.532
Stage 1	139(29.8)	131(28.1)	
Stage 2	40(8.6)	35(7.5)	
Stage 3	18(3.9)	13(2.8)	
In-hospital dialysis	13(2.8)	8(1.7)	0.377
RBC transfusion	196(42.1)	175(37.6)	0.181
DSWI	10(2.1)	7(1.5)	0.624
LCOS	21(4.5)	25(5.4)	0.650
IABP application	18(3.9)	19(4.1)	1
Longer-term			
Lost to follow-up	14(3.1)	10(2.2)	0.535
New onset of dialysis	5(1.1)	3(0.7)	0.708
Mortality	33(7.5)	37(8.3)	0.753

*MI* myocardial infarction, *AKI* acute kidney injury, *RBC* red blood cell, *DSWI* deep sternal wound infection, *LCOS* low cardiac output syndrome, *IABP* intra-aortic balloon pump

### Long-term outcomes

**Fig. 2:**
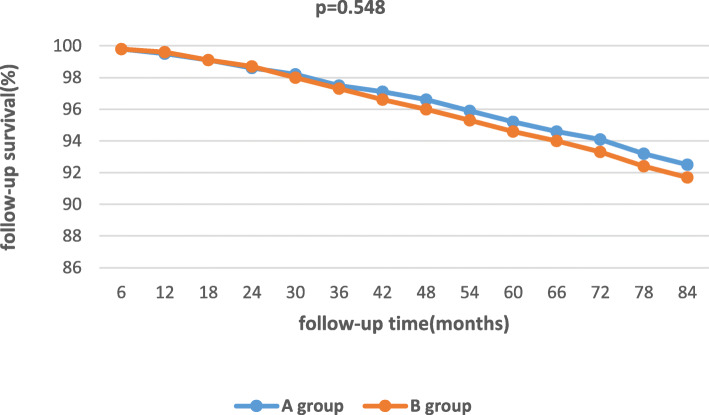
Actuarial curves of longer-term survival of the matched cohorts

**Fig. 3:**
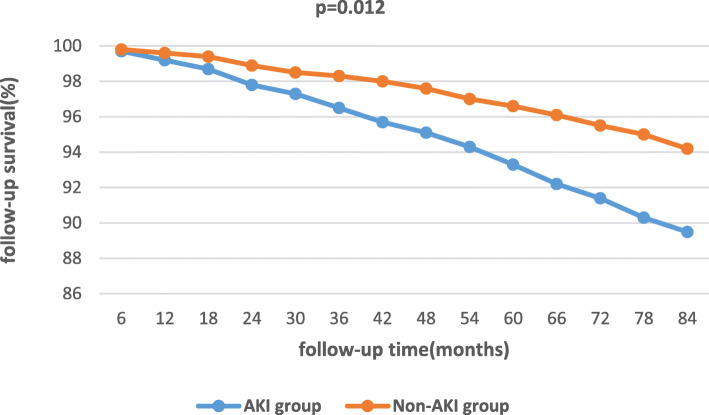
Actuarial curves of longer-term survival of the AKI group and non-AKI group

The mean follow-up time was 50.6 ± 9.5 months. There were 70 patients died during the follow-up (33 in A group and 37 in B group), and 24 patients were lost to follow-up totally(14 in A group and 10 in B group). (Table 2). Comparing with A group, B group did not have an decreased new onset of dialysis (χ2 = 0.14, *p* = 0.708) or mortality (χ2 = 0.099, *p* = 0.753) during the long-term follow-up(Table 2), (Fig. 2). Comparing with in-hospital non-AKI patients, in-hospital AKI patients were associated with an increased long-term new onset of dialysis (χ2 = 8.153, *p* = 0.004) and decreased long-term survival (χ2 = 6.277, *p* = 0.012). (Table 3), (Fig. 3). Multivariable logistic regression manifested that the HR for long-term new onset of dialysis and mortality in AKI patients vs. non-AKI patients was 1.83 and 1.31 respectively (95%CI 1.12–2.86, *p* = 0.007; 95%CI 1.17–2.58, *p* = 0.015). The HR for long-term new onset of dialysis and mortality in on-pump group vs. off-pump group was 1.07 and 1.11 respectively (95%CI 1.03–1.23, *p* = 0.661; 95%CI 1.09–1.64, *p* = 0.702). (Table 4).

**Table 3:** In-hospital and longer-term outcomes in AKI and non-AKI group

variable	AKI group	Non-AKI group	*p* value
	(*n* = 382) No. (%)	(*n* = 550) No. (%)	
In-hospital			
New onset of dialysis	21(4.5)	0	0.000
Mortality	12(3.1)	8(1.5)	0.129
Longer-term			
Lost to follow-up	11(2.9)	13(2.4)	0.780
New onset of dialysis	9(2.4)	1(0.2)	0.004
Mortality	39(10.5)	31(5.8)	0.012

**Table 4:** Predictors of long-term new onset of dialysis /long-term mortality

Variable	HR	95% CI	*p* value
In-hospital AKI vs. non-AKI	1.83/1.31	1.12–2.86/1.17–2.58	0.007/0.015
Age (per y)	1.25/1.79	1.13–1.72/1.27–2.02	0.003/<0.0001
Gender (Female vs. male)	1.24/1.47	1.03–1.55/1.03–2.02	0.21/0.19
DM	1.39/1.41	1.02–1.88/1.09–1.53	0.012/0.035
LVEF<0.50	1.43/1.98	1.02–2.27/1.05–2.26	0.025/0.003
On-pump vs. off-pump	1.07/1.11	1.03–1.23/1.09–1.64	0.661/0.702
IABP application	2.32/2.06	1.11–2.87/1.05–2.10	0.001/<0.0001

## Discussion

The principal findings of this single-centre, retrospective PSM study can be summarized as follows. (1) AKI of any severity occurred in 40.3% of all CABG patients aged older than 70 years, with AKIN stage 1 accounting for most AKI cases(71.8%). New onset of dialysis was applied in 2.3% of all patients. (2) Comparing with on-pump CABG, off-pump CABG was neither associated with decreased rate or severity of postoperative AKI nor with decreased long-term new onset of dialysis or mortality in such cohort. (3) Postoperative in-hospital AKI increased the rate of new onset of dialysis and decreased the survival rate in long-term follow-up.

PSM analysis provided the opportunity to rule out confusion by providing balanced baseline and procedural characteristics except the application of CPB. Certainly there was no significant difference in other factors, including the experience of surgeons. The univariate factor analysis manifested that the two PSM groups had similar in-hospital mortality and most of morbidities.

AKI occurs in up to 30% of patients undergoing CABG, depending on the underlying definition [13]. The rate of new onset of renal dialysis following cardiac surgery ranged from 1 to 6% [14]. Veterans Affairs Randomized On/Off Bypass (ROOBY) trial showed new renal dialysis within 30 days postoperatively was 0.9% [15]. Garg et al. performed a detailed analysis evaluating kidney function of the CORONARY patient population, overall 1.2% of these patients needed new renal dialysis [16, 17]. In our study, AKI was a common complication that occurred in 40% of all patients, and 2.3% needed renal dialysis. The incidence in our study was higher than that reported in the literatures. In addition, the incidence of new renal dialysis for AKI patients was 4.5% in our study, which was obviously higher than the average. The same result was obtained by Wilko Reents et al., they promoted a study named” The German Off Pump Coronary Artery Bypass Grafting in Elderly Patients (GOPCABE)”, which only enrolled patients aged 75 years or older to CABG with or without CPB [18]. It is therefore reasonable to assume that the relative high rate of AKI and new renal dialysis in our study was mainly due to the high proportion of particularly vulnerable elderly patients with preexisting kidney dysfunction, as was shown in table1, average baseline GFR was only about 81 ml/min/1.73m^2^. As is reported that age and preoperative renal dysfunction are both independent risk factors of AKI [19].

CPB still contributes to renal injury due to multiple perturbations in renal physiology and function as mentioned before. Off-pump technique seems to be a logical step toward preventing postoperative AKI. However, the effect of off-pump CABG on kidney function is still discussed controversially in literatures [14, 20]. The largest single randomized trial (CORONARY) found an absolute 4.1% risk reduction of AKI for off-pump CABG, but the reduction was contributed only by decreased occurrence of AKI stage 1, more severe stages of AKI or new renal dialysis were of no significant differences between the two groups, our center was also one of the collaborators of the CORONARY study [16]. A meta-analysis of 37 randomized controlled trials (*n* = 3449) and 22 risk-adjusted observational studies (*n* = 293,617) concluded that the benefit for off-pump CABG was only in the observational studies, but the difference was not significant in the aggregate randomized trials [21].

In this study we also found that compared with on-pump CABG, off-pump CABG was not associated with decreased rate or severity of AKI in elderly patients. We speculated the reasons were as follows: (1) The different definitions of AKI used in this study and methodological concerns precluded definitive conclusions from other trials. (2) Average CPB duration(mean time was 78.4 min) was relatively short, which attenuated pump-induced hemolysis, thereby releasing hemoglobin and free iron, and injuring the renal tubule [22]. (3) Relative high perfusion pressure was kept to guarantee the renal perfusion, especially in elderly patients undergoing on-pump surgery [23]. (4) The inflammatory response did not differ greatly between on-pump and off-pump groups [24, 25]. (5) When performing the anastomosis of the lateral vessels, transient circulatory failure and global hypoperfusion (renal malperfusion) often occurred during the off-pump surgery. (6) Atheroembolism caused by aortic manipulation(cross and side clamping) is another possible reason of renal impairment, which may occur in both on- and off-pump surgery. (7) On-pump technique provided improved complete revascularization, so it is conducive to the recovery of cardiac function and renal perfusion. (8) There are no active treatments for AKI, and therefore, perioperative preventative strategies seem particularly promising. Keep adequate hydration and avoid the use of diuretics; minimize the use of medications with adverse effects on renal function; keep an optimal hemodynamic status and correct the acid-base or metabolic imbalance. All of the aboved strategies might offset the differences in renal impairment between the two groups.

As expected, the follow-up demonstrated that in-hospital AKI patients showed a trend to higher new onset of dialysis (2.4% vs 0.2%, *p* = 0.004) and lower long-term survival (89.5% vs 94.2%, *p* = 0.012) compared with non-AKI patients in this study. Cox regression manifested that postoperative in-hospital AKI was a significant variable related to the new onset of dialysis and long-term mortality, and the HR was 1.83 and 1.31 respectively (95%CI 1.12–2.86, *p* = 0.007; 95%CI 1.17–2.58, *p* = 0.015). This study was consistent with our previous study and some other studies which demonstrated that development of AKI was associated with high short-term and long-term morbidity and mortality [18, 26]. AKI has been associated with progression to chronic kidney disease(CKD) and dialysis in many reports [27]. CKD and dialysis might exert negative efferts on the long-term survival inevitably especially in elderly patients [28]. Unfortunately, there are no pharmacologic agents known to reduce the risk of AKI or treat established AKI. Therefore, AKI patients after CABG need to strengthen the follow-up of nephropathy, more strictly management of the risk factors of coronary artery disease postoperatively.

### Limitations

Firstly, a retrospective, non-randomized single-centre analysis over a long period of time and with different surgeon’s procedures on patients undergoing CABG is subjected to the effects of selection bias, for example, low percentage of female patients and high percentage of patients with good ventricular function, baseline GFR was> 80%. Although PSM is implemented, a prospective, multi-centre study involving larger sample size is needed. Secondly, the GFR was derived by using the MDRD formula, which was not designed for determining GFR. Thirdly, the initial RIFLE criteria do not include the creatinine increase within 48 h for defining the mildest form of AKI, whereas the RIFLE categories injury and loss are similar to AKIN stage 2 and 3, respectively. AKI defined according to the RIFLE criteria may therefore avoid an iatrogenic hemodilution but may miss more subtle changes of kidney function [18]. Finally, on-pump CABG is usually associated with more grafts and more transfusions, but after PSM with off-pump group, some on-pump cases have to be excluded.

## Conclusions

In summary, this analysis revealed that AKI was common in elderly patients receiving CABG, but deterioration of dialysis was relatively scarce. We failed to observe better kidney function with off-pump vs. on-pump in terms of postoperative rate or severity of AKI in elderly patients. In-hospital AKI increased the rate of new onset of dialysis and decreased the survival rate in elderly patients in the longer-term.

## Data Availability

All data and material are available by contacting wr1582@163.com

## Abbreviations

PSM: Propensity score matching
CPB: Cardiopulmonary bypass
AKI: Acute kidney injury
CABG: Coronary artery bypass grafting
AKIN: Acute kidney injury network
GFR: Glomerular filtration rate
PS: Propensity score
BMI: Body mass index
DM: Diabetes mellitus
COPD: Chronic obstructive pulmonary disease
MI: Myocardial infarction
PCI: Percutaneous coronary intervention
LVEF: Left ventricular ejection fraction
LIMA: Left internal mammary artery
DSWI: Deep sternal wound infection
LCOS: Low cardiac output syndrome
IABP: Intra-aortic balloon pump
CKD: Chronic kidney disease
